# Colors, colored overlays, and reading skills

**DOI:** 10.3389/fpsyg.2014.00833

**Published:** 2014-07-29

**Authors:** Arcangelo Uccula, Mauro Enna, Claudio Mulatti

**Affiliations:** ^1^Dipartimento di Storia, Scienze dell’Uomo e della Formazione, Università degli Studi di SassariSassari, Italy; ^2^Dipartimento di Psicologia dello Sviluppo e della Socializzazione, Università degli Studi di PadovaPadova, Italy

**Keywords:** color, overlays, meares-irlen syndrome, reading, dyslexia

## Abstract

In this article, we are concerned with the role of colors in reading written texts. It has been argued that colored overlays applied above written texts positively influence both reading fluency and reading speed. These effects would be particularly evident for those individuals affected by the so called Meares-Irlen syndrome, i.e., who experience eyestrain and/or visual distortions – e.g., color, shape, or movement illusions – while reading. This condition would interest the 12–14% of the general population and up to the 46% of the dyslexic population. Thus, colored overlays have been largely employed as a remedy for some aspects of the difficulties in reading experienced by dyslexic individuals, as fluency and speed. Despite the wide use of colored overlays, how they exert their effects has not been made clear yet. Also, according to some researchers, the results supporting the efficacy of colored overlays as a tool for helping readers are at least controversial. Furthermore, the very nature of the Meares-Irlen syndrome has been questioned. Here we provide a concise, critical review of the literature.

## INTRODUCTION

The role of colors in reading has a few decades of history, dating back to 1958, when Jansky (1958) reported the case of a student with a reading deficit who was unable to recognize words printed on a white paper but was able to recognize words printed on a yellow paper. Although the theoretical debate on the causes of reading difficulties and dyslexia has given a primary role to the “phonological hypothesis” – since the efficiency of the processes of phonological processing is among the best predictors of reading skill acquisition (Wagner and Torgesen, 1987; Snowling et al., 2000) – the role of visual and perceptual skills has gained attention (e.g., Watson et al., 2003). One of the reasons that brought the role of visual and perceptual skills in reading to attention was the observation that some dyslexic individuals are affected by a perceptual dysfunction, called Scotopic Sensitivity Syndrome and also known as Meares-Irlen Syndrome and Visual Stress (MISViS; Evans, 1997).

In this article, we provide a brief, concise review of the literature on colored overlays as a remedy for visual stress in reading. To forecast the conclusions, the conception of visual stress as an independent reading deficit is controversial, whereas the research on the colored overlays is yet inconclusive since evidence both in favor of and contrary to their efficacy as a remedy has been provided.

## VISUAL STRESS AND READING

The term “visual stress” refers to the inability to see comfortably and without distortion (Wilkins et al., 1984). With “visual stress” Wilkins refers to the condition caused by the features of the visual stimulus, and that therefore is of sensorial origin, and not to the visual stress generated by movements of the eyes, by visual accommodation or by binocular convergence. Symptoms of visual stress are visual fatigue, perceived excessive luminosity, and several kinds of perceptual distortion such as blurring, fading, or flickering of the visual stimulus. According to Irlen (1997), this condition would interest approximately the 12–14% of the population and about the 46% of individuals with a diagnosis of dyslexia (and/or alternative learning difficulties). A more recent study (Kriss and Evans, 2005) suggests that visual stress affects about the 37.5% of children with dyslexia and about the 25% of non-dyslexic children. The frequencies of the symptoms would be: blurring (24%), duplication (16%), jump (12%), format switch (6%), and fading (3.5%) of the visual stimulus (Kriss and Evans, 2005).

According to Meares (1980), the factors that contribute most to the reading difficulties in children originates in the perceptual instability of the visual input due to the organization of the figure with respect to the background of the black ink writing on a white paper, which is typical in printed books. The idea, therefore, is that for some individuals the reflex of the black ink on a white paper makes reading difficult.

## COLORED OVERLAYS

The idea here is that if visual stress is the result of the relation between the visual features of black ink writing on white paper, then changing this relation might result in a reduction of the symptoms associated with visual stress (c.f. Wilkins, 2003; Irlen, 2010). A way to alter the relation between the visual features of the written text and the background is to place on the text a colored sheet of transparent plastic (colored overlay). Scott et al. (2002; see also Kruk et al., 2008) had shown that whereas bad readers show – after about 10 min of reading black writing on a white paper – the typical symptoms of visual stress, they do not show visual stress’ symptoms when reading the texts with the same characteristics through a colored overlay.

Thus, the implications of the colored overlays method is that if visual stress impairs reading acquisition, then the use of colored overlays might improve both reading and reading acquisition (Irlen, 2010).

## DYSLEXIA AND COLORED OVERLAYS

According to Evans et al. (1999) colored filters determines benefit in about 80% of individuals using them. The adoption of colored overlays/filters in schools is incremented given that the visual stress syndrome – which symptoms they are supposed to alleviate – is often observed in dyslexic students (Irlen, 1991; Singleton and Trotter, 2005; Singleton and Henderson, 2007), and it is in schools that students are usually diagnosed as dyslexics. The estimation of visual stress is, in fact, often included in tests aimed at assessing reading skills and dyslexia (Nichols et al., 2009), and the colored overlays are often used as a remedy for the visual stress symptoms co-occurring with dyslexia. However, several studies have shown that dyslexia and visual stress are independent conditions. Originally, in fact, visual stress was considered as a subset of dyslexia, whereas more recently it has been argued that the visual stress syndrome is independent from dyslexia (Kriss and Evans, 2005; Kruk et al., 2008). Indeed Kriss and Evans (2005) noted that the prevalence of visual stress in dyslexic individuals is of only 10% higher than in the non-dyslexic individuals: from this the authors conclude that dyslexia and visual stress are two independent conditions which sometimes coexist within the same individual.

Although dyslexia and visual stress seem independent syndromes, it is often the case that significantly large sub-groups of dyslexics do have deficits in visual processing (Watson and Willows, 1995), and when dyslexia is associated with a visual-perceptual deficit, reading difficulties worsen (Wilkins et al., 2001). In fact, it has been shown that when dyslexic children can read through a self-chosen colored overlay, they reading speed increases of about a 25% (Wilkins, 2002): moreover, although it seems that even non-dyslexic children benefit from the use of colored overlays, the benefit resulting from the use of colored overlays by dyslexic children is higher than that observed with non-dyslexic children (Singleton and Henderson, 2007). With respect to adults, it seems that only individuals with dyslexia and visual stress syndrome benefit from the use of colored overlays when compared with dyslexics without visual stress, non-dyslexics with visual stress, and non-dyslexics without visual stress.

Singleton and Trotter (2005) classified a sample of dyslexic and non-dyslexic individuals as a function of whether they experienced high or low intensities of visual stress, and observed that only the dyslexics individuals experiencing visual stress of high intensity benefitted from colored overlays. From this, the authors concluded that dyslexia and visual stress are related: they argued that if the two conditions were independent, as proposed by Wilkins, all individuals experiencing intense visual stress should have benefitted from colored overlays, regardless the concurrent presence of dyslexia. Noteworthy, the argument of Singleton and Trotter assumes that colored overlays were always beneficial for visual stress, when in presence of visual stress, and since colored overlay are not beneficial for not dyslexics individuals with intense visual stress, visual stress and dyslexia are inter-dependent. But of course, one could argue here that it is the efficacy of colored overlay that depends on the coexistence of the two conditions, regardless of whether or not the two conditions are dependent.

Thus, there exist two views. According to one view, visual stress and dyslexia are independent conditions. According to the other view, visual stress and dyslexia are dependent conditions.

## HOW DOES COLOR HELP READING (IF IT DOES)?

Despite the many studies aimed at investigating the role of colors in reading – also altering the features of the letters (Pinna et al., 2010) – and that the colored overlay are widely used, the mechanisms at the basis of the relation between reading and color have not been properly understood. Possibly, one of the reason for this lack of explanations is that the very nature of the visual stress syndrome and of its role in reading has been questioned, and therefore the entire enterprise might just be a false trail.

A recent account of the causes of visual stress posits that a strong sensorial stimulation – as a dense written text – might lead to a reduction of the efficiency of the inhibitory mechanisms in the visual cortex, thus resulting in an excessive excitation of the cortical neurons, and this would cause illusions and distortions (Huang et al., 2003). This hypothesis implies that some individuals are affected by a sort of cortical hypersensitivity so that their visual cortex would overreact to intense visual stimulations thus determining the symptoms associated with visual stress, as fatigue and migraine. Building on this ground, Wilkins and Evans (2010) proposed that the colored overlays are effective because they distribute this excessive excitation and thus mitigate the symptoms of visual stress, thus improving written text processing and reading. Although this account lacks of strong empirical evidence (Henderson et al., 2013), a recent neuroimaging study by Chouinard et al. (2012) provides some initial evidence showing cortical over-excitability in presence of visual stress syndrome.

This view of the basis of visual stress is congruent with early studies (Wilkins et al., 1994; Robinson and Foreman, 1999) showing that the color of the colored overlay is specific for each individual, that is whit the fact that each reader benefits from the use of colored overlays only if the color of the overlay is a specific color.

Some of the symptoms of visual stress as blurring and illusory migrations of letters are similar to those reported in presence of magnocellular dysfunctions (Stein and Walsh, 1997). A dysfunction of the magnocellular pathway would produce long lasting, anomalous visual traces which would interfere – by masking – with the visual processing of the stimulation thus causing blurring and distortions. The empirical evidence here is once more inconsistent (Skoyles and Skottun, 2009).

Wilkins (2003) argues that the hypothesis of a magnocellular dysfunction at the basis of visual stress might account for the individual differences in the use of colors – this because it has been shown that each individual benefits from the use of a given, specific color, not from any possible color. This last proposal lacks of empirical evidence.

According to some authors, the candidate brain structure for understanding the relation between colored overlays and reading is the magnocellular system (Chase et al., 2003). In fact, it has been shown that reading is compromised within a red light environment compared to a green light environment, this because the red light inhibits the activity of the magnocellular system (Chase et al., 2003). Similarly, Ray et al. (2005) have shown that yellow filters – by reducing the blue components of the light which inhibit the activity of the magnocellular system – increase the ability of reading in dyslexic populations (however, this has not been replicated, see: Palomo-Álvarez and Puell, 2013). Although these findings are consistent with the idea that reading proficiency benefits from the use of colored filters, they are inconsistent with early findings on colored overlays, since early findings show that each individual benefits from the use of a particular, given color, whereas these latter findings suggest that a particular color – e.g., yellow – should work for any reader.

## LATEST DEVELOPMENTS ON COLORED OVERLAYS: DO THEY WORK OR NOT?

In recent studies, serious methodological limits in the works supporting the use of colored overlays have been pointed out.

One of the main methodological issues has to do with the definition and the diagnosis of visual stress and originates in the way visual stress is assessed. Some authors diagnose or not visual stress as a function of how participants responds to treatments based on colored overlays (Kriss and Evans, 2005). Others instead stress the symptoms of visual stress as visual distortions in reading (Singleton and Trotter, 2005). It has been noted that in order to use the improvements in reading due to the use of colored overlays as a diagnostic criterion, the symptoms should be univocally attributable to the Meares-Irlen syndrome, which is not necessarily the case (Kruk et al., 2008). In addition, some have considered a 20% increase in reading speed with the use of colored overlays as a threshold for the diagnosis of visual stress (Minwook et al., 2014), others used a 5% increase in reading speed as criterion. Of course, the prevalence of the Meares-Irlen syndrome changes as a function of the threshold used. Wilkins et al. (2001) found that with a threshold of 5% of increase in reading speed due to colored overlays, the 33% of 6–8 years old children suffers of visual stress. With a threshold of 10%, the prevalence falls to 12.5% (Kriss and Evans, 2005), whereas with a threshold of 25%, the prevalence falls to 5% (Wilkins et al., 2001). The prevalence of visual stress increases if the samples are limited to dyslexic individuals, and goes from 47% with a threshold of 5–31% with a threshold of 10%.

Noteworthy, the evaluation of the symptoms is based on subjective reports, and, in the studies of Wilkins and colleagues (e.g., Wilkins et al., 2005), the participants select their favorite colors or combination of colors themselves. From one side, these aspects question the reliability of the diagnosis, as confirmed by low test-retest reliability (Woerz and Maples, 1997). From another side, these specificity and variability in the selection of the color complicate the search for an explanation of why a color is better than another for a given individual, especially assuming visual stress is a unique condition.

Some recent studies failed to find statistically significant effects of colored overlays. Ritchie et al. (2011) had shown that, in the short period, colored overlays do not speed reading up compared to non-colored overlays, whether or not the participants have a diagnosis of visual stress. Ritchie et al. (2012) had shown that – compared to a control condition – not even one year of use of colored overlays results in an increase in reading speed and accuracy. Henderson et al. (2013) had shown that despite the fact that often dyslexic individuals do experience stronger visual stress than controls, neither dyslexics nor controls benefit from the use of colored overlays.

## DISCUSSION AND CONCLUSION

The existence itself of the visual stress syndrome is – at least as an independent condition – controversial: symptoms that have been considered mapping into an independent cluster might just be individual-specific aspects of the more wide and articulated dyslexia. In addition, typical visual stress symptoms might be symptoms of dyslexia rather than causes (Olitsky and Nelson, 2003), and thus the attenuation of those symptoms – whatever the technique employed – might have no consequences on the quality of the reading. It has been shown that children with reading difficulties do like to play video games and do play video games for long time: some have argued that if at the basis of their reading difficulties there were perceptual deficits, then they would avoid such high intensity visual activities as video gaming [American Academy of Pediatrics (AAP), 2009]. However, it has been shown that playing action video games improves reading skills of dyslexic children more than traditional reading treatments, possibly because action video games improve attentional abilities (Franceschini et al., 2013). This implies that despite their lower attentional abilities, dyslexic children do like to play video games and, also, obtain benefits from playing video games. Thus, if the visual stress existed, then – analogously – children with visual stress might not only like to play video games, they might also obtain benefits from playing video games.

The idea at the basis of the Meares-Irlen syndrome, whether or not the syndrome exists as an independent collection of symptoms, contributed – by focusing on the early, input processes – to the identification of visual disorders which have been observed to occur in presence of reading difficulties or dyslexia, thus contrasting the dominant view of dyslexia that sees the deficit as due to phonological processing impairments (Ramus, 2014). For example, in a recent, single-case study of a dyslexic children, the authors found visual processing disorders but not phonological disorders (Valdois et al., 2011).

Whether colored overlays help reading or not seems at least controversial: although initial evidence was indeed provided, more recent studies both highlight the methodological issue of previous studies and show that colored overlays do not help reading (Ritchie et al., 2011, Ritchie et al., 2012; Henderson et al., 2013), On the ground of contradictory findings as these, the [American Academy of Pediatrics (AAP), 2009] has claimed that there is not empirical evidence toward the efficacy of colored overlays in reading, reading acquisition, or dyslexia, and did not recommend their use.

However, the participants in the studies of Ritchie et al. (2011); Ritchie et al. (2012) were non-dyslexic children, and in the study of Henderson et al. (2013) they were adults, whereas it has been shown that effects of colored overlays are more easily found with dyslexic children (Singleton and Trotter, 2005; Singleton and Henderson, 2007). Whether or not, at least in some conditions, colored overlay works does not seem to be a settled issue. Thus, although from one side, given these contradictory findings, a precautionary, prudent position – as that of the Academy of Pediatrics – on the use of colored overlay seems desirable, especially in clinical or educative contexts, from another side, given that some evidence that the colored overlays work exists, concluding that colored overlays proved not worth in allaying reading problems is premature and, possibly, incorrect.

## Conflict of Interest Statement

The authors declare that the research was conducted in the absence of any commercial or financial relationships that could be construed as a potential conflict of interest.
